# Educational and economic determinants of food intake in Portuguese adults: a cross-sectional survey

**DOI:** 10.1186/1471-2458-4-58

**Published:** 2004-12-02

**Authors:** Pedro A Moreira, Patricia D Padrão

**Affiliations:** 1 Faculty of Nutrition and Food Sciences, University of Porto, R. Roberto Frias, 4200-465 Porto, Portugal

## Abstract

**Background:**

Understanding the influences of educational and economic variables on food consumption may be useful to explain food behaviour and nutrition policymaking. The aim of this study was to evaluate the importance of educational and economic factors in determining food pattern in Portuguese adults.

**Methods:**

A cross-sectional study in a representative sample of Portuguese adults (20977 women and 18663 men). Participants were distributed in four categories according to years of education (≤4, 5–9, 10–12, and >12) and income (≤314 euros, 315–547 euros, 548–815 euros, and >815 euros). Logistic regression models were fitted to estimate the magnitude of the association between food groups and education/income, adjusting for confounders.

**Results:**

In both genders, the odds favouring milk, vegetable soup, vegetables, fruit, and fish consumption, increased significantly with education, for those having >12 years of education compared to those with ≤4 years; the odds favouring wine, and spirits consumption decreased significantly with education, for those having >12 years of education compared to those with ≤4 years. In males, the odds favouring starchy foods and meat consumption decreased significantly with income, while for milk, the odds increased with higher income (those having >815 euros compared to those with ≤314 euros).

**Conclusions:**

The low and high income groups are or tend to be similar in regard to several food groups consumption, and access to education/information appears to be the key element to a better food pattern as indicated by higher frequency of milk, vegetable soup, vegetables, fruit, and fish consumption.

## Background

There is a large published literature on associations between socio-economic position and chronic disease, with socioeconomically disadvantaged groups experiencing higher mortality and morbidity rates for coronary heart disease, noninsulin dependent diabetes mellitus and some cancers [1-4]. Chronic diseases are largely preventable diseases, and diet has been known fore many years to play a key role as a risk factor for chronic diseases. While age, sex and genetic susceptibility are non-modifiable, many of the risks associated with age and sex are modifiable. Such risks include a complex mixture of interacting socio-economic, cultural and other environmental factors [5-8].

The relationship between socio-economic factors and diet has been examined on the basis of food and nutrient intake and the results are mixed, sometimes contradictory, and often the observed differences are small [9,10]. When differences are found, it is usually the case that persons from socioeconomically disadvantage backgrounds have food intakes consistent with their higher rates of chronic diseases [11-17].

In the context of European countries, economic development and increased purchasing power have recently changed the food availability situation [18]. On the other hand, socio-cultural influences may contribute, along with economic constraints, to particular food choices, which may explain the still substantial differences in food consumption across European countries [19]. Understanding the influences of socio-economic variables on food consumption may be useful to predict the outcome of interventions, to change food behaviour, and generate hypotheses concerning food consumption in diverse circumstances, as well as to explain observations in epidemiological studies.

The aim of this study was to evaluate the importance of educational and economic factors in determining food choice in a representative sample of the Portuguese general adult population.

## Methods

### Subjects and general characteristics

Data for this study were drawn from the Portuguese third National Health Survey (National Health Systems Observatory, National Institute of Health – Dr. Ricardo Jorge, Ministry of Health) carried out in 1998–1999. The study sample included all subjects (20977 women and 18663 men) older than 18 years, who reported their education level, income, physical activity, smoking habits, weight, height, and food intake when participating in the National Health Survey. Subjects were selected from 21808 households distributed according to the five regions of Portugal (there are five regions in mainland Portugal, namely Norte, Centro, Lisboa/Vale do Tejo, Alentejo, and Algarve; these regions are the portuguese NUTS II subdivisions), using a multi-stage random probability design. This probabilistic sample is representative of the Portuguese population from the Continental area (Azores and Madeira islands were not included). The survey response rate was 82%.

Trained interviewers conducted face-to-face interviews with the person in each household and inquired participants on social and demographic characteristics, smoking status (non-smokers, ex-smokers, smoking less than one cigarette per day, and smoking one or more cigarettes per day), weight, height (those anthropometric measures were self-reported and body mass index – BMI – was then calculated), food and beverages intake, and daily physical activity (occupational and leisure-time physical activity).

### Physical activity

Occupational physical activity was measured using the respondent's own occupation at the time of the survey. Respondents were asked about what best characterized their daily occupational activity, namely: usually seated and walking during short periods of time; standing activities or walking during long periods of time without carrying loads to often; carrying light objects or walking upstairs/downstairs several times; heavy physical work or carrying heavy objects; or don't know.

Respondents were asked to describe their leisure-activity using the following classification: heavy training and competitive sports more than once a week; running or practicing recreational sports or gardening activities ≥4 hours per week; walking for pleasure, bicycling (light effort) or doing other light activities ≥4 hours per week; reading, watching television or other sedentary activities; and don't know. Respondents were also asked to provide information about whether they had regular activities (once or more per week) such as running or bicycling (enough to make them feel tired).

### Food and beverages intake

Respondents were asked twelve questions related to their intake of central food groups and beverages, namely milk, vegetable soup, meat, fish, vegetables, fruit, bread, starchy foods (pasta/rice/potatoes), beer, spirits, Port Wine, and wine, and the consumption was recorded as a yes (when the respondent indicated the consumption of the food) or no answer. Because the data were collected by interviewers within the framework of an epidemiological study that was not specifically designed to assess quantitative aspects of nutritional and food intake, the dietary assessment method employed generic classifications of food groups, rather than specific varieties or species (fish rather than fatty fish or salmon, etc.), or quantitative measures. Consumption of these food items was determined by asking "For each of the listed food items please indicate those consumed": "during the day before the interview" (vegetable soup, meat, fish, vegetables, fruit, bread, and starchy foods – pasta, rice and potatoes); "during the week before interview" (beer, spirits, and Port Wine); and "daily consumed" (milk and wine).

### Education

Respondents were asked to provide information about whether they had attained further education since leaving school and if so, the highest qualification completed. Respondent's education was subsequently classified in four levels of education: less than 4 years, 5–9 years, 10–12 years, and more than 12 years.

### Income

Respondent's were asked to estimate the total income (including pensions, allowances and investments) received by all household members in the last month and to indicate this using a single measure comprising ten narrow-ranged income categories. This measure was subsequently re-coded into four categories according the number of salaries: less than 315 euros, 315–547 euros, 548–815 euros, and >815 euros.

### Statistical analysis

Separate logistic regression models were fitted for male and female to estimate the magnitude of the association between food groups consumption and education or income categories, adjusting for age, BMI, smoking habits, physical activity and income/education. An exploratory approach was chosen in the selection of explanatory variables in order to control for as many potentially significant variables as possible in the regression model. The choice of variables (age, BMI, smoking habits and physical activity) was based on findings reported in the literature, our own experience with specifically Portuguese factors associated with food consumption and their associations with the variables of interest; education was also adjusted for income and vice versa.

Student's t-tests, ANOVA, Spearman rank correlation analyses and chi-squared tests were used to compare BMI, age, frequency of smoking, physical activity categories between genders to determine the degree to which those variables correlated with education and income. A *p*-value of less than 0.05 was considered statistically significant. Statistics were performed using SPSS 12.0.

## Results

The study sample comprised 20977 women (52,9%) and 18663 men, with mean ages of 50.3 (±18.88) and 47.7 (±18.51) years, respectively; BMI was significantly lower in women than in men (25.1 ± 4.53 Kg/m^2 ^*versus *25.6 ± 3.83, p < 0.001). There was a lower proportion of smokers among women compared to men (8.2% *versus *30.5%, p < 0.001). General characteristics (gender, age, BMI, smoking status, and physical activity) by education and income categories are presented in Tables 1 and 2.

**Table 1:** Characteristics of Portuguese adults by education categories

	Education			
	
	≤4 years	5–9 years	10–12 years	>12 years
Gender				
Female	54.0%	22.8%	12.6%	10.6%
Male	49.7%	28.7%	13.2%	8.4%
Age (years)				
Female	55.6 ± 14.47	37.8 ± 14.44	31.7 ± 13.63	34.7 ± 13.50
Male	55.3 ± 14.72	37.0 ± 14.99	33.0 ± 14.44	38.7 ± 16.19
BMI (Kg/m^2^)				
Female	26.4 ± 4.45	24.3 ± 4.03	22.5 ± 3.51	22.3 ± 3.45
Male	26.3 ± 3.91	25.1 ± 3.63	24.5 ± 3.52	24.6 ± 3.19
Smokers				
Female	3.1%	16.1%	19.1%	18.7%
Male	26.0%	42.9%	32.6%	29.2%
Physical activity in females				
Daily occupational activity best characterized by				
Usually seated, walking short periods of time	29.2%	28.8%	30.2%	30.4%
Standing, walking long periods of time	44.5%	46.1%	41.7%	42.0%
Carrying light objects, walking up/downstairs	11.3%	11.0%	12.2%	11.1%
Heavy physical work or carrying heavy objects	14.9%	14.1%	15.8%	16.5%
Don't know	0.1%	0%	0.1%	0.1%
Leisure-activity best characterized by				
Heavy training/competitive sports (>1x/wk)	3.4%	3.3%	3.6%	3.1%
Running/recreational sports/gardening (≥4 h/wk)	9.3%	7.8%	9.0%	9.7%
Walking for pleasure, bicycling light (≥4 h/wk)	20.4%	21.2%	19.3%	21.2%
Reading, watching TV, sedentary activities	66.8%	67.7%	68.0%	66.0%
Don't know	0.1%	0%	0.1%	0%
Regular activity such as running or bicycling, enough to feel tired				
Yes	13.1%	11.9%	13.2%	14.2%
No	86.8%	88.1%	86.7%	85.8%
Don't know	0.1%	0%	0.1%	0%
Physical activity in males				
Daily occupational activity best characterized by				
Usually seated, walking short periods of time	36.4%	37.3%	37.5%	36.2%
Standing, walking long periods of time	51.6%	51.5%	50.9%	50.0%
Carrying light objects, walking up/downstairs	8.3%	8.2%	7.5%	10.5%
Heavy physical work or carrying heavy objects	3.6%	3.0%	3.9%	3.3%
Don't know	0.1%	0%	0.2%	0%
Leisure-activity best characterized by				
Heavy training/competitive sports (>1x/wk)	0.9%	0.5%	1.1%	1.2%
Running/recreational sports/gardening (≥4 h/wk)	5.1%	4.6%	4.1%	4.9%
Walking for pleasure, bicycling light (≥4 h/wk)	15.9%	15.7%	16.8%	16.7%
Reading, watching TV, sedentary activities	78.1%	79.1%	77.8%	77.2%
Don't know	0.1%	0.1%	0.2%	0%
Regular activity such as running or bicycling, enough to feel tired				
Yes	7.0%	6.0%	6.7%	7.2%
No	92.9%	94.0%	93.2%	92.8%
Don't know	0.1%	0.0%	0.2%	0%

**Table 2:** Characteristics of Portuguese adults by income categories

	Income			
	
	≤314 euros	315–547 euros	548–815 euros	>815 euros
Gender				
Female	16.3%	25.4%	24.5%	33.8%
Male	20.8%	24.3%	22.8%	32.2%
Age (years)				
Female	50.7 ± 18.97	50.3 ± 18.90	50.1 ± 18.68	50.2 ± 18.94
Male	47.9 ± 18.54	47.2 ± 18.45	47.6 ± 18.44	48.1 ± 18.55
BMI (Kg/m^2^)				
Female	25.3 ± 4,55	25.0 ± 4.51	25.1 ± 4.52	25.1 ± 4.55
Male	25.6 ± 3.70	25.4 ± 3.95	25.6 ± 3.74	25.7 ± 3.88
Smokers				
Female	7.4%	8.0%	8.6%	8.2%
Male	28.5%	31.9%	30.3%	30.7%
Physical activity in females				
Daily occupational activity best characterized by				
Usually seated, walking short periods of time	30.3%	26.0%	24.6%	35.7%
Standing, walking long periods of time	46.4%	43.8%	42.8%	43.6%
Carrying light objects, walking up/downstairs	10.3%	11.4%	13.5%	10.0%
Heavy physical work or carrying heavy objects	12.7%	18.7%	19.0%	10.6%
Don't know	0.2%	0%	0.1%	0.1%%
Leisure-activity best characterized by				
Heavy training/competitive sports (>1x/wk)	1.2%	2.3%	3.1%	5.4%
Running/recreational sports/gardening (≥4 h/wk)	4.0%	7.3%	11.1%	11.1%
Walking for pleasure, bicycling light (≥4 h/wk)	16.7%	19.2%	19.0%	24.0%
Reading, watching TV, sedentary activities	77.9%	71.2%	66.7%	59.5%
Don't know	0.1%	0%	0.1%	0.1%
Regular activity such as running or bicycling, enough to feel tired				
Yes	5.8%	9.1%	12.0%	19.1%
No	94.1%	90.9%	87.9%	80.8%
Don't know	0.1%	0%	0.1%	0.1%
Physical activity in males				
Daily occupational activity best characterized by				
Usually seated, walking short periods of time	41.8%	33.5%	32.2%	39.3%
Standing, walking long periods of time	48.1%	53.2%	53.1%	50.8%
Carrying light objects, walking up/downstairs	7.6%	9.6%	10.0%	7.0%
Heavy physical work or carrying heavy objects	2.4%	3.6%	4.7%	2.8%
Don't know	0.1%	0.1%	0%	0%
Leisure-activity best characterized by				
Heavy training/competitive sports (>1x/wk)	0.3%	0.5%	0.8%	1.4%
Running/recreational sports/gardening (≥4 h/wk)	2.9%	4.4%	5.5%	5.4%
Walking for pleasure, bicycling light (≥4 h/wk)	10.3%	13.1%	16.3%	21.5%
Reading, watching TV, sedentary activities	86.4%	81.9%	77.4%	71.7%
Don't know	0.1%	0.2%	0%	0%
Regular activity such as running or bicycling, enough to feel tired				
Yes	2.2%	4.7%	5.4%	11.7%
No	97.6%	95.2%	94.6%	88.3%
Don't know	0.1%	0.1%	0%	0%

In women, the odds favouring milk, vegetable soup, vegetables, fruit, and fish consumption, increased with increasing education (p-values for trends were always ≤0.046), being the odds ratios, respectively, 2.60 (2.24–3.01), 1.20 (1.05–1.38), 1.75 (1.44–2.13), 1.92 (1.49–2.49), and 1.40 (1.23–1.60) for those having >12 years of education compared to those with ≤4 years, after adjusting for age, BMI, smoking habits, physical activity and income (Tables 3 and 4). The odds favouring bread, starchy foods (other than bread), wine, and spirits consumption in women decreased with increasing education (p trend ≤ 0.002), being the odds ratios, respectively, 0.44 (0.34–0.56), 0.68 (0.53–0.87), 0.51 (0.41–0.62), and 0.13 (0.03–0.53) for those having >12 years of education compared to those with ≤4 years (Tables 3 and 4).

**Table 3:** Odds ratios for food consumption according level of education, adjusted for age, BMI, smoking habits, physical activity and income

**Women**				**Men**			
	**OR**	**IC(95%)**	**P trend**		**OR**	**IC(95%)**	**P trend**

**Vegetable soup**				**Vegetable soup**			
Education				Education			
≤4 years		(reference)		≤4 years		(reference)	
5–9 years	0.90	0.82–1.00		5–9 years	0.99	0.90–1.09	
10–12 years	0.94	0.83–1.07		10–12 years	1.07	0.94–1.21	
>12 years	1.20	1.05–1.38	0.046	>12 years	1.15	1.00–1.32	0.045
**Vegetables**				**Vegetables**			
Education				Education			
≤4 years		(reference)		≤4 years		(reference)	
5–9 years	1.05	0.92–1.20		5–9 years	1.09	0.97–1.23	
10–12 years	1.17	0.99–1.39		10–12 years	1.38	1.17–1.62	
>12 years	1.75	1.44–2.13	<0.001	>12 years	1.44	1.19–1.74	<0.001
**Fruit**				**Fruit**			
Education				Education			
≤4 years		(reference)		≤4 years		(reference)	
5–9 years	1.30	1.09–1.55		5–9 years	1.30	1.13–1.49	
10–12 years	1.63	1.29–2.06		10–12 years	1.75	1.45–2.13	
>12 years	1.92	1.49–2.49	<0.001	>12 years	1.68	1.35–2.10	<0.001
**Bread**				**Bread**			
Education				Education			
≤4 years		(reference)		≤4 years		(reference)	
5–9 years	0.78	0.63–0.96		5–9 years	0.97	0.75–1.25	
10–12 years	0.50	0.39–0.64		10–12 years	0.73	0.54–1.00	
>12 years	0.44	0.34–0.56	<0.001	>12 years	0.44	0.33–0.59	<0.001
**Other starchy**				**Other starchy**			
Education				Education			
≤4 years		(reference)		≤4 years		(reference)	
5–9 years	0.99	0.81–1.20		5–9 years	1.09	0.88–1.35	
10–12 years	0.72	0.57-0.92		10-12 years	1.07	0.80-1.43	
>12 years	0.68	0.53-0.87	<0.001	>12 years	1.15	0.83-1.60	0.355
**Fish**				**Fish**			
Education				Education			
≤4 years		(reference)		≤4 years		(reference)	
5-9 years	1.06	0.96-1.17		5-9 years	1.14	1.04-1.25	
10-12 years	1.24	1.09-1.40		10-12 years	1.36	1.20-1.54	
>12 years	1.40	1.23-1.60	<0.001	>12 years	1.50	1.31-1.72	<0.001
**Meat**				**Meat**			
Education				Education			
≤4 years		(reference)		≤4 years		(reference)	
5-9 years	1.01	0.89-1.14		5-9 years	1.09	0.96-1.23	
10-12 years	1.02	0.86-1.21		10-12 years	1.27	1.06-1.52	
>12 years	0.95	0.80-1.13	0.693	>12 years	1.16	0.96-1.41	0.014

**Table 4:** Odds ratios for beverage consumption according level of education, adjusted for age, BMI, smoking habits, physical activity and income

**Women**				**Men**			
	**OR**	**IC(95%)**	**P trend**		**OR**	**IC(95%)**	**P trend**

**Milk**				**Milk**			
Education				Education			
≤4 years		(reference)		≤4 years		(reference)	
5-9 years	1.54	1.38-1.70		5-9 years	1.53	1.39-1.68	
10-12 years	2.24	1.95-2.57		10-12 years	3.00	2.62-3.44	
>12 years	2.60	2.24-3.01	<0.001	>12 years	3.07	2.62-3.59	<0.001
**Wine**				**Wine**			
Education				Education			
≤4 years		(reference)		≤4 years		(reference)	
5-9 years	0.79	0.68-0.93		5-9 years	0.73	0.63-0.84	
10-12 years	0.45	0.36-0.55		10-12 years	0.41	0.35-0.49	
>12 years	0.51	0.41-0.62	<0.001	>12 years	0.46	0.38-0.56	<0.001
**Beer**				**Beer**			
Education				Education			
≤4 years		(reference)		≤4 years		(reference)	
5-9 years	0.92	0.72-1.18		5-9 years	0.86	0.77-0.97	
10-12 years	0.76	0.56-1.03		10-12 years	0.61	0.52-0.71	
>12 years	0.82	0.61-1.10	0.097	>12 years	0.57	0.48-0.68	<0.001
**Spirits**				**Spirits**			
Education				Education			
≤4 years		(reference)		≤4 years		(reference)	
5-9 years	0.66	0.27-1.58		5-9 years	0.72	0.60-0.87	
10-12 years	0.15	0.03-0.74		10-12 years	0.46	0.34-0.62	
>12 years	0.13	0.03-0.53	0.002	>12 years	0.27	0.19-0.40	<0.001
**Port Wine**				**Port Wine**			
Education				Education			
≤4 years		(reference)		≤4 years		(reference)	
5-9 years	1.44	1.03-2.01		5-9 years	1.00	0.81-1.24	
10-12 years	1.22	0.80-1.88		10-12 years	0.97	0.73-1.30	
>12 years	1.34	0.90-2.00	0.265	>12 years	1.42	1.07-1.89	0.093

In men, similar odds ratios were observed for milk, vegetable soup, vegetables, fruit, fish, bread, wine, and spirits (Tables 3 and 4). However, in men but not in women, odds favouring meat consumption increased with increasing education (OR = 1.16 (0.96–1.41) for those having >12 years of education compared to those with ≤4 years), while for beer consumption, odds decreased with increasing education (OR = 0.57 (0.48–0.68) for those having >12 years of education compared to those with ≤4 years).

No such significant trends were observed for these food groups and income with the exceptions of meat and starchy foods (other than bread) consumption, in men, which decreased with increasing income (p trend ≤ 0.022), and milk consumption which increased with increasing income (Tables 3 and 4).

## Discussion

The main finding of the present study is that educational attainment was more frequently associated with food choices than income. There is general agreement among researchers [20-23] that education and income are conceptually distinct, and that they are likely to make separate and unique contributions to health-related outcomes [24]. In our study, the most educated consumed more frequently fruit, vegetables, milk and fish, and less wine and spirits, than their counterparts from less educated groups.

Over the last years, several studies have attempted to identify the influence of socioeconomic factors on individual's dietary intake [25-28]. Our interest in educational and economic determinants of food choice in Portuguese adults relate to these particular characteristics in the population. Portugal, according European standards, is a small and relatively poor country, exhibiting the highest level of social inequalities in the European Union [29]. Nevertheless, Portugal had significantly and positively changed in the last four decades, in several domains such as the economy and culture, although the census of 1991 revealed that 15.3% of the Portuguese were illiterate. That of 2000 showed that, despite the improvements and changes in the education of adults, 7% can still not read or write [29]. This is a reality that classifies Portugal as the country with the higher percentage of individuals with low level of education in all the European Union [29]. From the employment perspective, Portugal's unemployment rates in the last 25 years never surpassed 10% of the active population, which is a better indicator than the observed levels in the majority of the European countries. However, the percentage of individuals with low-remuneration in Portugal is much higher than the EU average [29].

Several studies have concluded that a strong relationship exists between countries' per capita national incomes and nutrition [30-32]. The economic issue is of considerable significance, and it is sometimes suggested that this is probably the key variable of all in influencing food choice [30]. Household income is expected to influence food choices, especially for relatively high-priced food items such as fish, fresh fruit and vegetables [33]. Nevertheless, this not seems to be the case when we compared income and education levels as determinants of intake of significant food groups in Portuguese adults. Our data shows, in both genders, a significant positive trend in the consumption of vegetables, vegetable soup, fruits, milk and fish, with higher levels of education, which did not occurred in relation to income with the exception of milk. In our study, education was adjusted for income and vice versa. While the majority of investigators use two or more indicators of socio-economic position, several [34-37] do not simultaneously adjust for the unmeasured effects of each indicator on the other. Two types of bias may result from this practice: (1) using a single indicator such as education may bias the point estimate (food choice) because the education variable is allowed to account for some of the variation that is actually the product of unmeasured socio-economic influences; as a result, if we did not simultaneously adjusted education for income and vice versa, our claims about the influence of education level on food choice probably would have been overestimated; (2) the use of a single indicator may result in the overall or total socio-economic effect being underestimated.

Data from the Portuguese Household Budget Surveys (using the DAta Food Networking – DAFNE – classification system), shows similar results to ours in relation to the positive association between education attainment and the availability of fruits, fish, milk and alcoholic beverages but some different data in regard to other foods (availability of vegetables and cereal products is fairly stable or tends to decline with education) [18]. Curiously, we found that meat consumption in men was positively associated with level of education, as in the DAFNE study [18], although our study showed a significant reduction of meat consumption with higher categories of income, in men. In Portugal [18] fish is more available among the trend-leading educated individuals which may be more advantageous to their cardiovascular health [38].

In our study, there was also a significant trend in the consumption of milk in men, being more frequently ingested with increasing income. As suggested by Axelson, [39] positive health relationships between dietary patterns and income may reflect a growing concern about health in the higher socio-economic groups.

The association between milk consumption and socio-economic position is sometimes contradictory. Cristofar and Basiotis [40], for example, reported lower intake of milk among low-income women, while Roos et al. [41] found that higher educational and income groups from both genders consumed less milk.

Consumption of alcoholic beverages, such as wine and spirits, in both genders, and beer in men, exhibited significant decreases in their frequency of intake, with increasing education levels. In Portugal, alcoholic beverages consumption is a major public health problem [42]. In DAFNE study, [18] using Portuguese Household Budget Surveys, alcoholic beverages availability was also higher in the lower educated households.

Interestingly, in our study, the consumption of bread (in both genders) and other starchy foods (in women), decreased with increasing number of years of education; men seem to abandon starchy foods (other than bread) consumption under condition of higher incomes. It is possible that higher educated individuals tend to avoid foods that are considered as being more fattening or rich in energy, such as bread and other starchy foods [43,44]. Research has demonstrated that for a given body size, higher educated women are more dissatisfied with or concerned about their bodies and are more likely to have dieted in the past than lower educated women [45,46].

One of the most interesting findings in terms of economic constraints and food consumption relationship in our study, is the few significant associations between income and food choices, even though the well established links between economic and material resources, food availability and dietary quality [47]. By contrast to our results, Turrell et al. [23] showed household income to be the strongest and most robust independent predictor of food purchasing behaviour, and the effects of education to be substantially attenuated (to non-significance or marginal significance). In our study, the specificity of the relationship between education and food choice probably reflect each respondent's individual contribution to food choice, whereas household income was possibly capturing the combined contextual effects of numerous individuals, as well as many other within-household processes [23], and thus showed a weaker relationship with food choice. Our results may also reflect lesser difficulties faced by low-income groups when selecting the food groups that we studied. In several urban and rural areas of Portugal, there are many people who own plots of land that are too small to make a living, but allow them to work the land and produce foods (e.g., fruit, vegetables and poultry) for their own consumption. Although they produced a limited range of foods that is not accounted in official agricultural statistics, probably, if they stopped working the land they would experience greater difficulties in obtaining access to those particular foods. A potential limitation of our study and most nationwide population surveys is that the poor are usually not well presented. We know from previous research into survey participation that population-based samples typically under-represent the most socio-economically disadvantaged and over-represent the advantaged [48,49], because homeless and unemployed may be difficult to reach, and this may debilitates the interpretation of our results.

In our study, it remains to be explained the different pattern of associations between income and important food groups (milk versus fruit and vegetables, for example) and the different pattern of associations between food choices and income in each gender (e.g., milk and starchy foods). Several reasons may explain specific differences in the findings of our study compared with those of previous mentioned studies, including differences in populations sampled (e.g., both genders versus women only, different cultural backgrounds ranges), differences in assessment of education or income, differences in dietary assessment (e.g., qualitative food data versus 24-h dietary recalls or food frequency questionnaires) and differences in analytic methods (e.g., covariates included in statistical models). Nonetheless, results from our study indicate that the associations between food choice were stronger in relation to educational attainment than income categories. Differences in food choices according the level of education reflect that more knowledge may influence the perceived relationship between diet and health as well as the perceived outcomes of following a healthy diet [33].

Despite differences in food consumption according education and income, in our study we could not assess if these differences were also evident on the energy and nutrient level, which was a limitation. British data point to micronutrient and antioxidant intakes as the most likely nutritional influences on health inequalities [50]. Nevertheless, according Galobardes et al., [10] it is also possible that despite differences in food consumption, nutrient intake is similar among socio-economic groups, as these may not be substantial enough to translate into differences in nutrient intake.

If a country like Portugal wants to change the adult food choice behaviour, or in other words, wants to reach certain dietary goals, the support of applied research like ours is needed in order to plan the right strategies for promoting healthy diets. Confidence in a significant positive causal link between per capita national income and individual nutrition reinforces the importance of economic growth [51] but also implies that public policy should stress education as a mean for improving healthy food choices. Education might influence food choice by facilitating or constraining one's ability to understand the information communicated in nutrition education or in food labels [52,53].

Whereas income-related dietary differences suggest ameliorative responses through the potential of the economic system, differences based on education point to initiatives such as nutrition education programmes [22,54]. According to Geraldes,[55]in Portugal it may sometimes be more appropriate to correct inequalities in the domains of education or nutrition than that of health. Given the poor education level of the majority of Portuguese adults, a move towards an increased acquisition of general knowledge and personal development through compulsory and higher education, lifelong learning and improved qualifications of the population, is desirable to promote the development of a knowledge society and improve the level and quality of national education which, in turn, may relate to healthier food choices. It is well recognized that changes in dietary behaviour may be brought about, not by direct modification of food habits, but by alteration or manipulation of the education and culture [8].

## Conclusions

Regardless of the reasons explaining the complex and diversified patterns of economic and educational associations of food consumption found in Portugal, the findings of this study suggest that education and income have distinct associations with food choice. The low and high income groups are or tend to be similar in regard to the majority of food choices, and access to education appears to be the key element to a better food pattern as indicated by higher frequency of milk, vegetable soup, vegetables, fruit, and fish consumption.

## Competing interests

The author(s) declare that they have no competing interests.

## Authors' contributions

PM and PP designed the study. PM and PP did the statistical analysis, and PM wrote the paper. PM and PP reviewed the final version of the paper.

**Table 5:** Odds ratios for food consumption according level of income, adjusted for age, BMI, smoking habits, physical activity and education

**Women**				**Men**			
	**OR**	**IC(95%)**	**P trend**		**OR**	**IC(95%)**	**P trend**

**Vegetable soup**				**Vegetable soup**			
Income				Income			
≤314 euros		(reference)		≤314 euros		(reference)	
315-547 euros	1.02	0.91-1.14		315-547 euros	0.95	0.85-1.05	
548-815 euros	1.01	0.90-1.14		548-815 euros	0.95	0.85-1.06	
>815 euros	0.96	0.86-1.07	0.281	>815 euros	0.92	0.83-1.02	0.195
**Vegetables**				**Vegetables**			
Income				Income			
≤314 euros		(reference)		≤314 euros		(reference)	
315-547 euros	1.11	0.96-1.29		315-547 euros	1.22	1.06-1.39	
548-815 euros	1.07	0.92-1.24		548-815 euros	1.16	1.01-1.33	
>815 euros	1.05	0.92-1.21	0.768	>815 euros	1.06	0.93-1.20	0.796
**Fruit**				**Fruit**			
Income				Income			
≤314 euros		(reference)		≤314 euros		(reference)	
315-547 euros	0.94	0.81-1.21		315-547 euros	0.99	0.84-1.15	
548-815 euros	0.98	0.80-1.19		548-815 euros	0.95	0.81-1.12	
>815 euros	1.01	0.84-1.23	0.983	>815 euros	1.03	0.89-1.20	0.769
**Bread**				**Bread**			
Income				Income			
≤314 euros		(reference)		≤314 euros		(reference)	
315-547 euros	1.16	0.92-1.47		315-547 euros	1.05	0.80-1.39	
548-815 euros	1.06	0.84-1.34		548-815 euros	1.05	0.79-1.40	
>815 euros	1.00	0.81-1.25	0.502	>815 euros	0.85	0.66-1.10	0.139
**Other starchy**				**Other starchy**			
Income				Income			
≤314 euros		(reference)		≤314 euros		(reference)	
315-547 euros	0.86	0.69-1.06		315-547 euros	0.91	0.71-1.17	
548-815 euros	0.95	0.77-1.19		548-815 euros	0.84	0.65-1.07	
>815 euros	0.88	0.72-1.08	0.466	>815 euros	0.72	0.57-0.90	0.002
**Fish**				**Fish**			
Income				Income			
≤314 euros		(reference)		≤314 euros		(reference)	
315-547 euros	0.86	0.77-0.96		315-547 euros	0.99	0.89-1.10	
548-815 euros	0.84	0.75-0.93		548-815 euros	0.95	0.85-1.06	
>815 euros	0.92	0.83-1.03	0.409	>815 euros	0.98	0.88-1.08	0.457
**Meat**				**Meat**			
Income				Income			
≤314 euros		(reference)		≤314 euros		(reference)	
315-547 euros	0.98	0.86-1.12		315-547 euros	0.95	0.82-1.09	
548-815 euros	1.09	0.95-1.25		548-815 euros	0.92	0.80-1.06	
>815 euros	1.04	0.92-1.19	0.299	>815 euros	0.87	0.76-0.99	0.026

**Table 6:** Odds ratios for beverage consumption according level of income, adjusted for age, BMI, smoking habits, physical activity and education

**Women**				**Men**			
	**OR**	**IC(95%)**	**P trend**		**OR**	**IC(95%)**	**P trend**

**Milk**				**Milk**			
Income				Income			
≤314 euros		(reference)		≤314 euros		(reference)	
315-547 euros	0.94	0.83-1.05		315-547 euros	1.01	0.90-1.12	
548-815 euros	1.02	0.91-1.15		548-815 euros	1.05	0.94-1.18	
>815 euros	0.99	0.89-1.11	0.575	>815 euros	1.12	1.01-1.24	0.017
**Wine**				**Wine**			
Income				Income			
≤314 euros		(reference)		≤314 euros		(reference)	
315-547 euros	0.97	0.81-1.17		315-547 euros	1.07	0.93-1.22	
548-815 euros	0.92	0.76-1.11		548-815 euros	1.00	0.87-1.14	
>815 euros	0.86	0.72-1.03	0.065	>815 euros	0.89	0.79-1.01	0.757
**Beer**				**Beer**			
Income				Income			
≤314 euros		(reference)		≤314 euros		(reference)	
315-547 euros	0.89	0.67-1.19		315-547 euros	0.95	0.83-1.09	
548-815 euros	0.89	0.66-1.20		548-815 euros	1.05	0.92-1.21	
>815 euros	1.00	0.76-1.31	0.671	>815 euros	1.06	0.93-1.21	0.139
**Spirits**				**Spirits**			
Income				Income			
≤314 euros		(reference)		≤314 euros		(reference)	
315-547 euros	2.18	0.74-6.43		315-547 euros	1.14	0.92-1.41	
548-815 euros	2.52	0.86-7.43		548-815 euros	1.12	0.89-1.40	
>815 euros	1.28	0.44-3.78	0.968	>815 euros	1.09	0.88-1.34	0.229
**Port Wine**				**Port Wine**			
Income				Income			
≤314 euros		(reference)		≤314 euros		(reference)	
315-547 euros	1.15	0.78-1.70		315-547 euros	0.98	0.77-1.24	
548-815 euros	1.28	0.87-1.89		548-815 euros	1.20	0.94-1.53	
>815 euros	1.09	0.75-1.59	0.704	>815 euros	1.05	0.84-1.33	0.483

## Pre-publication history

The pre-publication history for this paper can be accessed here:


